# Assessment of multi-modality evaluations of obscure gastrointestinal bleeding

**DOI:** 10.3748/wjg.v23.i4.614

**Published:** 2017-01-28

**Authors:** Ryan Law, Jithinraj E Varayil, Louis M WongKeeSong, Jeff Fidler, Joel G Fletcher, John Barlow, Jeffrey Alexander, Elizabeth Rajan, Stephanie Hansel, Brenda Becker, Joseph J Larson, Felicity T Enders, David H Bruining, Nayantara Coelho-Prabhu

**Affiliations:** Ryan Law, Jithinraj E Varayil, Louis M WongKeeSong, Jeffrey Alexander, Elizabeth Rajan, Stephanie Hansel, Brenda Becker, David H Bruining, Nayantara Coelho-Prabhu, Division of Gastroenterology and Hepatology, Mayo Clinic, Rochester, MN 55905, United States; Jeff Fidler, Joel G Fletcher, John Barlow, Division of Radiology, Mayo Clinic, Rochester, MN 55905, United States; Joseph J Larson, Felicity T Enders, Division of Health Sciences Research, Mayo Clinic, Rochester, MN 55905, United States

**Keywords:** Double balloon enteroscopy, Computed tomography enterography, Video capsule enteroscopy, Obscure gastrointestinal bleeding

## Abstract

**AIM:**

To determine the frequency of bleeding source detection in patients with obscure gastrointestinal bleeding (OGIB) who underwent double balloon enteroscopy (DBE) after pre-procedure imaging [multiphase computed tomography enterography (MPCTE), video capsule endoscopy (VCE), or both] and assess the impact of imaging on DBE diagnostic yield.

**METHODS:**

Retrospective cohort study using a prospectively maintained database of all adult patients presenting with OGIB who underwent DBE from September 1^st^, 2002 to June 30^th^, 2013 at a single tertiary center.

**RESULTS:**

Four hundred and ninety five patients (52% females; median age 68 years) underwent DBE for OGIB. AVCE and/or MPCTE performed within 1 year prior to DBE (in 441 patients) increased the diagnostic yield of DBE (67.1% with preceding imaging *vs* 59.5% without). Using DBE as the gold standard, VCE and MPCTE had a diagnostic yield of 72.7% and 32.5% respectively. There were no increased odds of finding a bleeding site at DBE compared to VCE (OR = 1.3, *P* = 0.150). There were increased odds of finding a bleeding site at DBE compared to MPCTE (OR = 5.9, *P* < 0.001). In inpatients with overt OGIB, diagnostic yield of DBE was not affected by preceding imaging.

**CONCLUSION:**

DBE is a safe and well-tolerated procedure for the diagnosis and treatment of OGIB, with a diagnostic yield that may be increased after obtaining a preceding VCE or MPCTE. However, inpatients with active ongoing bleeding may benefit from proceeding directly to antegrade DBE.

**Core tip:** The yield of double balloon enteroscopy (DBE) without preceding video capsule endoscopy (VCE) or multiphase computed tomography enterography (MPCTE) was 59.4%, and with preceding imaging was 67.5%. Overall diagnostic yield of antegrade DBE is superior to CTE and equivalent to VCE in the evaluation of obscure gastrointestinal bleeding. The diagnostic yields of DBE for inpatients *vs* outpatients were similar but the highest sensitivity of VCE using DBE as gold standard was in inpatients (84.9%). The incremental diagnostic yield of DBE of all patients with negative preceding VCE and MPCTE was 66% (35/53 patients). An appropriate strategy might be antegrade DBE in inpatients with evidence of ongoing bleeding if DBE is available.

## INTRODUCTION

Obscure gastrointestinal bleeding (OGIB) is defined as bleeding from the gastrointestinal (GI) tract that persists or recurs without an obvious etiology after negative upper endoscopy and colonoscopy[1,2]. OGIB is further categorized into “obscure overt” or “obscure occult” bleeding based on presence or absence of evident bleeding and accounts for approximately 5% of all GI bleeding[3]. Though lesions may be missed in the esophagus, stomach, and colon, the etiology of OGIB is secondary to small bowel pathology in up to 75% of cases[4-6] leading some experts to recommend that this term be replaced by the term ‘small bowel bleeding’[7]. The evaluation of OGIB frequently requires significant utilization of resources and results in patient frustration due to lack of definitive findings and clinical improvement in many cases[8].

The current American Society of Gastrointestinal Endoscopy guidelines recommend a variety of diagnostic options when evaluating OGIB, with slight differences between the “overt” and “occult” GI bleeding algorithms[9]. At many referral centers, multiphase computed tomography enterography (MPCTE) and/or video capsule endoscopy (VCE) are performed after a negative routine endoscopic exam but prior to double balloon enteroscopy (DBE) as these diagnostic studies are less invasive and may direct DBE-guided therapies[10,11]. MPCTE allows for evaluation of dynamic changes in abnormal enhancement patterns and compares findings across phases in 2-dimensional and 3-dimensional images[12]. Images are evaluated in arterial, enteric and delayed phases allowing for evaluation of ongoing or recent bleeding. In contrast, VCE requires ingestion of a small pill-size camera that provides endoluminal photographs of the entire GI tract for evaluation of small bowel mucosal lesions. These technologies are generally considered complimentary as each can provide different but vital information in the evaluation of OGIB. Though generally performed before DBE, there is a paucity of data regarding how often these tests alter subsequent diagnostic evaluation or treatment.

In this study, we aimed to determine the frequency of bleeding source detection in patients with OGIB who underwent DBE (antegrade/retrograde) after pre-procedure imaging (*i.e*. MPCTE, VCE, or both) and to assess the impact of imaging on DBE diagnostic yield. We also aimed to assess the agreement between findings of the pre-procedure imaging and the DBE itself.

## MATERIALS AND METHODS

A retrospective cohort study was conducted following the approval of the Institutional Review Board of Mayo Clinic-Rochester (IRB No. 13-002000 and No. 14-009997). Medical records were reviewed of all adult patients presenting with OGIB who underwent a DBE (antegrade/retrograde) from September 1^st^, 2002 to June 30^th^, 2013 using a prospectively maintained DBE database. Patients who underwent DBE for indications other than OGIB (*i.e*. enteral feeding tube placement, failed colonoscopy, evaluation for hereditary polyposis syndromes, small bowel mass, strictures, *etc*.) were excluded. The electronic medical record was utilized to obtain demographic, endoscopic, radiologic, and clinical outcomes data. At our institution, single balloon enteroscopy is not utilized for assessment of OGIB, and hence this procedure was not included in our study.

Demographic features including age at the time of DBE procedure, gender, and gastrointestinal surgeries prior to DBE were recorded. The total number of blood transfusions up to 30 d prior to the date of DBE procedure and the use of anticoagulant/antiplatelet agents at the time of procedure were collected. Details of VCE and MPCTE performed prior to the DBE were also collected. Only VCE and MPCTE performed within 1 year prior to the DBE procedure were included. All VCE were performed using Pillcam or Pillcam2 (Given Imaging, Yoqneam, Israel). When VCE was performed prior to DBE the date of the procedure, positive and negative findings, and time from VCE to DBE were noted. Positive VCE findings were categorized as (1) arteriovenous malformation (AVM); (2) red spot; (3) frank blood; (4) polyp; (5) ulcer; or (6) other[13]. Similarly, when MPCTE was performed prior to DBE, positive or negative findings and time from MPCTE to DBE were recorded. Positive MPCTE findings were categorized as (1) vascular malformations; (2) blood; (3) polyp/tumor; (4) ulcer; and (5) other[14,15]. Cross-sectional imaging findings were abstracted from the final radiologic report.

DBE procedural details included the approach (antegrade *vs* retrograde), type of obscure bleeding (overt *vs* occult), and hospital admission status (inpatient *vs* outpatient). The OGIB was defined as “overt” when the clinician’s note reported it to be overt or when there was clinically-evident bleeding including melena or hematochezia reported in the medical records. OGIB was defined as “occult” when the clinician’s review reported it to be occult, or when iron deficiency anemia or positive stool testing for blood loss were the sole indication for DBE. We documented whether total enteroscopy was achieved, defined as complete evaluation of the small bowel using either a single approach or combined antegrade-retrograde approach. Findings from DBE were classified into (1) vascular lesions (angioectasias/AVMs, Dieulafoy’s lesion, or ectopic varices); (2) mucosal lesions [erythema, erosions, ulcers, inflammation]; or (3) tumor/polyp[16]. If none of the above findings were seen, then the DBE was reported as negative. Therapies performed including argon plasma coagulation (APC), biopsy, hemostatic clip placement, bipolar cauterization, polypectomy and stricture dilation were also recorded. Adverse events including bleeding within 7 d of the procedure, perforation, pancreatitis and re-bleeding within 1 year of the procedure were recorded. Any repeat DBE performed within 1 year of the index DBE was documented.

### Statistical analysis

The statistical methods of this study were reviewed by Joseph Larson and Felicity Enders, PhD from the Mayo Clinic Division of Health Sciences Reserch. Continuous measures were summarized using medians and ranges while categorical measures were summarized using counts and percentages. Differences among two groups were assessed using the Kruskall-Wallis test and Chi-square or Fisher’s exact test for continuous or categorical measures, respectively.

To evaluate the predictive ability of VCE and MPCTE to identify bleeding sites, DBE was treated as the gold standard and the sensitivity, specificity, diagnosis yield, and accuracy were calculated among patients with VCE and MPCTE within one year of DBE. Ninety-five percent confidence intervals for each of these measures were also determined. This analysis was repeated among the following subgroups; antegrade and retrograde approach, inpatient and outpatient procedure, overt and occult bleeding.

Because the same patients underwent VCE or MPCTE and DBE, to assess the findings from the procedures, matched logistic regression performed with the finding treated as the outcome and the DBE test treated as the predictor. Odds ratios and 95% confident intervals along with *P* values are presented for these tests.

All analyses used an significance level of 5% and were performed using the SAS (v9.3, SAS Institute Inc., Cary, NC, United States).

## RESULTS

During the study period, 495 patients [51.5% females; median age 68.2 (range: 18.1-95.4) years] underwent DBE for OGIB. Overt OGIB was reported in 253 (51.1%) patients, and occult OGIB was reported in 242 (48.9%) patients. The procedure was performed in an outpatient setting in 381 (77.0%) patients and in an inpatient setting in 114 (23.0%) patients. The type of DBE approach was antegrade in 331 (75.1%) patients and retrograde in 110 (24.9%) patients. Total enteroscopy was achieved in a bidirectional manner in 19 (4.3%). Additional demographic data including DBE cases with surgically-altered anatomy and the use anticoagulant/antiplatelet agents is noted in Table 1.

**Table 1:** Patient characteristics *n* (%)

	**DBE negative**	**DBE positive**	**Total**	***P* value**
	**(*n* = 164)**	**(*n* = 331)**	**(*n* = 495)**	
Age at DBE, median (range)	65.0 (18.1-95.4)	69.5 (18.3-91.8)	68.2 (18.1-95.4)	0.005^1^
Gender				0.104^2^
Male	71 (43.3)	169 (51.1)	240 (48.5)	
Female	93 (56.7)	162 (48.9)	255 (51.5)	
Altered anatomy				0.736^2^
None	125 (76.2)	249 (75.2)	374 (75.6)	
Roux-en-Y	13 (7.9)	21 (6.3)	34 (6.9)	
Billroth/Ileo-colonic/IPAA	7 (4.3)	21 (6.3)	28 (5.7)	
Other	19 (11.6)	40 (12.1)	59 (11.9)	
On warfarin	19 (11.6)	36 (10.9)	55 (11.1)	0.813^2^
On clopidogrel	7 (4.3)	20 (6.0)	27 (5.5)	0.413^2^
On ASA 325	49 (29.9)	118 (35.6)	167 (33.7)	0.201^2^
VCE performed within 1 yr prior to DBE	47 (29.4)	130 (40.9)	177 (36.9)	
MPCTE performed within 1 yr prior to DBE	31 (19.4)	53 (16.7)	84 (17.5)	
VCE and MPCTE performed within 1 yr prior to DBE	67 (41.9)	113 (35.5)	180 (37.8)	
DBE performed without VCE and MPCTE done	15 (9.4)	22 (6.9)	37 (7.7)	
Type of OGIB				0.728^2^
Overt	82 (50.0)	171 (51.7)	253 (51.1)	
Occult	82 (50.0)	160 (48.3)	242 (48.9)	
Type of approach of DBE				< 0.001^2^
Anterograde	103 (62.8)	268 (81.0)	371 (74.9)	
Retrograde	61 (37.2)	63 (19.0)	124 (25.1)	
Total enteroscopy done	9 (5.5)	15 (4.5)	24 (4.8)	0.641^2^
Procedure location				0.393^2^
Inpatient	34 (20.7)	80 (24.2)	114 (23.0)	
Outpatient	130 (79.3)	251 (75.8)	381 (77.0)	

1 Kruskal Wallis;

2 χ^2^. VCE: Video capsule endoscopy; MPCTE: Multiphase computed tomography enterography; DBE: Double balloon enteroscopy; IPAA: Ileal pouch-anal anastomosis; OGIB: Obscure gastrointestinal bleeding.

Of the 495 patients, 458 patients had had VCE and/or MPCTE performed prior to DBE (441 patients within 1 year prior to DBE). Of the 441 patients, 296 had a positive DBE finding (yield of 67.1%). The findings noted on DBE in these patients are outlined in Table 2. The remaining 37 patients underwent a DBE without a preceding VCE or MPCTE. Of these 37 patients, 22 had a positive finding (yield of 59.5%, *P* = 0.36).

**Table 2:** Double balloon enteroscopy findings, therapy and complications *n* (%)

	**DBE negative**	**DBE positive**	**Total**
	**(*n* = 145)**	**(*n* = 296)**	**(*n* = 441)**
DBE findings			
Angioectasia/arterio venous malformation	0 (0.0)	212 (71.6)	212 (48.1)
Dieulafoy lesion	0	8 (2.7)	8 (1.8)
Varix	0	3 (1.0)	3 (0.7)
Evidence of Crohn’s disease	0	5 (1.7)	5 (1.1)
Erythema	0	19 (6.4)	19 (4.3)
Erosion	0	31 (10.1)	31 (6.8)
Ulcer	0	56 (18.9)	56 (12.7)
Polyp identified	0	50 (16.9)	50 (11.3)
Other findings	40 (27.6)	64 (21.6)	104 (23.6)
Therapy and complications			
Any therapy done?	4 (2.8)	296 (100)	300 (68.0)
Epinephrine injection	1 (0.7)	2 (0.7)	3 (0.7)
Biopsy	3 (2.1)	89 (30.1)	92 (20.9 )
Clipping	0	67 (22.6)	67 (15.2)
Argon plasma coagulation	0	213 (72.0)	213 (48.3)
Bipolar cauterization	0	10 (3.4)	10 (2.3)
Early re-bleeding (< 24 h)	0	7 (2.4)	7 (1.6)
Late re-bleeding (24 h-1 yr)	4 (2.8)	14 (4.7)	18 (4.1)
Pancreatitis	0	1 (0.3)	1 (0.2)

VCE: Video capsule endoscopy; MPCTE: Multiphase computed tomography enterography; DBE: Double balloon enteroscopy.

Among the 441 patients with VCE and/or MPCTE prior to DBE, therapeutic or diagnostic applications were performed in 300 (68.0%) patients including APC in 213 (48.3%) patients, biopsy in 92 (20.9%) patients, hemostatic clip placement in 67 (15.2%) patients, and bipolar cauterization in 10 (2.3%) patients. Early rebleeding (< 24 h from the time of procedure) was reported in 7 (2.4%) patients when the DBE was positive. Late rebleeding (24 h - 1 year from the time of index DBE) was reported in 14 (4.7%) patients when DBE findings were positive and 4 (2.8%) patients when DBE findings were negative (Table 2). A single patient developed pancreatitis and there were no perforations as complications.

Among 337 patients who had a VCE performed within the year preceding DBE, a bleeding site was identified at VCE in 171 (73.4%) patients when the DBE was positive and in 74 (71.2%) patients when the DBE was negative (*P* = 0.692). The median number of days between VCE and DBE was not significantly different when the DBE was positive (42; range: 0-356) or negative (35; range: 0-351) (*P* = 0.924) (Table 3). Using DBE as the gold standard, VCE had a sensitivity of 73.4% (95%CI: 67.7%-79.1%), specificity of 28.8% (95%CI: 20.1%-37.6%), and diagnostic yield of 72.7% (95%CI: 67.9%-77.5%) (Table 4). Among the patients with negative DBE, the commonest VCE findings were AVM (26.0%), blood (22.1%) and ulcer (16.3%).

**Table 3:** Video capsule endoscopy and multiphase computed tomography enterography findings of all patients who had a video capsule endoscopy and multiphase computed tomography enterography performed within 1 year prior to double balloon enteroscopy *n* (%)

	**Capsule endoscopy**				**Computed tomography enterography**			
	**DBE negative**	**DBE positive**	**Total**	***P* value**	**DBE negative**	**DBE positive**	**Total**	***P* value**
	**(*n* = 104)**	**(*n* = 233)**	**(*n* = 337)**		**(*n* = 95)**	**(*n* = 157)**	**(*n* = 252)**	
Capsule endoscopy positive	74 (71.2)	171 (73.4)	245 (72.7)	0.692^2^				
MPCTE positive					27 (28.4)	55 (35.0)	82 (32.5)	0.332^2^
Days from VCE to DBE, median (range)	42 (0-356)	35 (0-351)	37 (0-356)	0.924^1^				
Days from MPCTE to DBE, median (range)					19 (0-338)	29 (0-351)	23.5 (0-351)	0.162^1^
VCE and DBE within 30 d of each other	41 (39.4)	105 (45.1)	146 (43.3)	0.344^2^				
MPCTE and DBE within 30 d of each other					59 (62.1)	84 (53.5)	143 (56.7)	0.192^2^
Arterio-venous malformation	27 (26.0)	94 (40.3)	121 (35.9)	0.014^2^				
Vascular lesion					13 (48.1)	32 (58.2)	45 (54.9)	0.108^2^
Blood	23 (22.1)	51 (21.9)	74 (22.0)	0.999^2^				
Red spot	13 (12.5)	18 (7.7)	31 (9.2)	0.220^2^				
Polyp	4 (3.8)	12 (5.2)	16 (4.7)	0.784^2^				
Ulcer	17 (16.3)	20 (8.6)	37 (11.0)	0.040^2^	2 (7.4)	1 (1.8)	3 (3.7)	
Other					2 (7.4)	0 (0.0)	2 (2.4)	

1 Kruskal Wallis;

2 Fisher exact. VCE: Video capsule endoscopy; MPCTE: Multiphase computed tomography enterography; DBE: Double balloon enteroscopy.

**Table 4:** Comparison of video capsule endoscopy and multiphase computed tomography enterography in all patients with capsule endoscopy and computed tomography enterography performed within 1 year of double balloon enteroscopy

**Comparison test**	**Statistic**	**Count summary**	**%**	**95%CI**	
				**Lower limit (%)**	**Upper limit (%)**
VCE	Total, *n*	337			
	Sensitivity	171/233	73.4	67.7	79.1
	Specificity	30/104	28.8	20.1	37.6
	DX yield	245/337	72.7	67.9	77.5
	Accuracy	201/337	59.6	54.4	64.9
MPCTE	Total, *n*	252			
	Sensitivity	55/157	35.0	27.6	42.5
	Specificity	68/95	71.6	62.5	80.6
	DX yield	82/252	32.5	26.8	38.3
	Accuracy	123/252	48.8	42.6	55.0

VCE: Video capsule endoscopy; MPCTE: Multiphase computed tomography enterography.

In 252 patients who had MPCTE in the year preceding DBE, a bleeding site was identified in 55 (35.0%) patients when the DBE was positive and in 27 (28.4%) patients when the DBE was negative. The median number of days between MPCTE to DBE was not significantly different when the DBE was positive (19.0; range: 0-338) or negative (29; range: 0-351) (*P* = 0.162) (Table 3). Using DBE as the gold standard, MPCTE had a sensitivity of 35.0% (95%CI: 27.6%-42.5%), specificity of 71.6% (95%CI: 62.5%-80.6%), and diagnostic yield of 32.5% (95%CI: 26.8%-38.3%) (Table 4). Among patients with negative DBE, the most common MPCTE findings were vascular lesions (48.1%), blood (33.3%), and ulcer (7.4%).

Of 53 patients who had preceding negative test(s), 35 (66.0%) had a positive DBE. Of these with positive DBE, 28 (80.0%) were antegrade DBE, 16 (45.7%) were for overt bleeding, and only 4 (11.4%) were in inpatients. AVMs were the commonest finding, found in 23 (65.7%) patients and treated with APC.

Of the 37 patients who went straight to DBE without preceding CE or MPCTE, the DBE was done antegrade in 26 (70.3%) patients, in 13 (35.1%) inpatients, and for overt bleeding in 26 (70.3%) patients. The commonest findings at DBE were AVM in 17 (45.9%) and ulcer in 8 (21.6%).

In order to compare findings on DBE to VCE and MPCTE, matched odds ratios were examined. There were no increased odds of finding a bleeding site at DBE compared to VCE (OR = 1.3, 95%CI: 0.9-1.7, *P* = 0.150). There were increased odds of finding a bleeding site at DBE compared to MPCTE (OR = 5.9, 95%CI: 3.5-9.7, *P* < 0.001).

When comparing by DBE approach, VCE had a diagnostic yield of 75.6% (95%CI: 70.3%-80.9%) with an antegrade approach; and a diagnostic yield of 63.9% (95%CI: 53.5%-74.2%) with a retrograde approach. MPCTE had a diagnostic yield of 31.6% (95%CI: 24.9%-38.2% with the antegrade approach and diagnostic yield of 35.4% (95%CI: 23.8%-47.0%) with a retrograde approach (Supplementary Table 1).

When comparing by procedure setting, VCE had a diagnostic yield of 76.7% (95%CI: 67.0%-86.4%) in the inpatient setting and a diagnostic yield of 71.6% (95%CI: 66.2%-77.0%) in the outpatient setting. With the inpatient setting, MPCTE had a diagnostic yield of 40.8% (95%CI: 27.1%-54.6%) and a diagnostic yield of 30.5% (95%CI: 24.2%-36.9%) with the outpatient setting (Supplementary Table 2). Thus, in the inpatient setting, VCE had higher diagnostic yield than MPCTE.

When comparing overt to occult bleeding, VCE had a diagnostic yield of 72.9% (95%CI: 66.3%-79.6%) for overt bleeds and a diagnostic yield of 72.5% (95%CI: 65.7%-79.2%) for occult bleeds. With overt bleeds, MPCTE had a diagnostic yield of 34.4% (95%CI: 26.0%-42.9%) and a diagnostic yield of 30.8% (95%CI: 22.8%-38.7%) with occult bleeds (Supplementary Table 3).

## DISCUSSION

Small bowel bleeding is the commonest cause of OGIB, seen in 75% of cases[17]. Identifying the site of bleeding and its therapy remain challenging due to this anatomic location. DBE is an effective way to address these challenges but is costly and not readily available at all centers. Our study aimed to look at the diagnostic yield of DBE and of preceding VCE and MPCTE. This would allow us to analyze the need for imaging prior to DBE. In our large single center cohort of 495 patients with OGIB, the yield of DBE without preceding VCE or MPCTE was 59.4%, and with preceding imaging was 67.5%. Thus, although the diagnostic yield of DBE is higher when pre-DBE imaging is positive, a source lesion is frequently identified when pre-DBE imaging is negative or not performed.

Using direct visualization by DBE as the gold standard, VCE had a diagnostic yield of 72.7% but a relatively low specificity of 28.8%. This is similar to prior studies[18]. The commonest findings at VCE with negative DBE were AVMs and blood; it is possible that these abnormalities had subsided by the time of the DBE since the time interval between the tests in our study could be up to 1 year[11,19]. This would be characteristic of AVMs which often bleed intermittently, and could artificially increase the apparent false positive rate.

A preceding MPCTE was done in fewer patients compared to VCE and had a lower diagnostic yield of 32.5%. However, the specificity was higher at 71.6%, also similar to previous studies[14,20,21]. The vascular lesions seen in nearly half the patients with positive MPCTE and negative DBE were likely deep in the bowel wall and hence not seen endoscopically.

Antegrade DBEs overall had higher diagnostic yields than retrograde DBEs (72.51% *vs* 50.91%, *P* < 0.001. This has been shown in one other smaller series[22]. Thus, the overall diagnostic yield of antegrade DBE is superior to CTE and roughly equivalent to VCE in the evaluation of OGIB. This is an important finding because it suggests that almost all patients should undergo antegrade DBE before retrograde, unless otherwise dictated by abnormal MPCTE suggesting ileal tumors or polyps.

The diagnostic yields of DBE for inpatients *vs* outpatients were similar in our data but the highest sensitivity of VCE using DBE as gold standard was in inpatients (84.9%). This group also showed the highest specificity (45.0%). Interestingly the incremental diagnostic yield of DBE of all patients with negative preceding VCE and MPCTE was 66% (35/53 patients). Thus, this raises the question of whether an appropriate strategy might be to directly proceed to antegrade DBE in inpatients with evidence of ongoing bleeding if DBE is available. This is also reflected by the matched odds ratios comparing VCE and MPCTE to DBE where there were no increased odds of finding a bleeding site at DBE compared to VCE and increased odds at DBE compared to MPCTE. In our data, none of the tests had a significantly higher yield in patients with overt bleeding compared to occult bleeding, which is unlike prior studies[23-25]

In conclusion, our data suggest that DBE is a generally safe and well tolerated procedure for the diagnosis and treatment of OGIB, with a diagnostic yield that may be increased after obtaining a preceding VCE or MPCTE. However, inpatients with active ongoing bleeding may benefit from proceeding directly to antegrade DBE, which has the benefits of improved diagnostic yield in these patients, ability to intervene therapeutically, and avoidance of an additional diagnostic test. A prospective evaluation and cost-effectiveness analysis of this clinical algorithm would be warranted.

## COMMENTS

### Background

The etiology of obscure gastrointestinal bleeding (OGIB) is secondary to small bowel pathology in up to 75% of cases. The authors sought to determine the frequency of bleeding source detection in patients with OGIB who underwent double balloon enteroscopy (DBE) after pre-procedure imaging (multiphase computed tomography enterography, video capsule endoscopy, or both) and assess the impact of imaging on DBE diagnostic yield.

### Research frontiers

Diagnostic yields of DBE, computed tomography enterography and video capsule enteroscopy in obscure gastrointestinal (GI) bleeding.

### Innovations and breakthroughs

This is the one of the largest cohort of patients with occult GI bleeding undergoing DBE for occult GI bleeding. A large proportion of patients also had preceding imaging, allowing for comparison of the various techniques.

### Applications

Inpatients with active ongoing bleeding may benefit from proceeding directly to antegrade DBE without preceding testing, which has the benefits of improved diagnostic yield in these patients, ability to intervene therapeutically, and avoidance of an additional diagnostic test.

### Terminology

Obscure GI bleeding - GI bleeding where etiology is not in the esophagus, stomach, or colon.

### Peer-review

Even though the theme has been studied for years allover the world, it is still necessary to update and clarify the best approach to a challenging clinical entity such as OGIB. This study is uptodate and follows the most recent guidelines and studies.
